# Contralateral acupuncture for migraine without aura: a randomized trial protocol with multimodal MRI

**DOI:** 10.3389/fnins.2024.1344235

**Published:** 2024-03-15

**Authors:** Ziwen Chen, Qifu Li, Yi Lu, Gaoyangzi Huang, Ya Huang, Xianmei Pei, Yi Gong, Bingkui Zhang, Xin Tang, Zili Liu, Taipin Guo, Fanrong Liang

**Affiliations:** ^1^College of Acupuncture and Tuina, Chengdu University of Traditional Chinese Medicine, Chengdu, China; ^2^School of Second Clinical Medicine/The Second Affiliated Hospital, Yunnan University of Chinese Medicine, Kunming, China; ^3^Department of Medical Imaging, The First Affiliated Hospital of Kunming Medical University, Kunming, China; ^4^Kunming Psychiatry Hospital/Yunnan University of Chinese Medicine Teaching Hospital, Kunming, China

**Keywords:** contralateral acupuncture, ipsilateral acupuncture, clinical trial, female, migraine without aura, multimodal MRI, brain functional connectivity, study protocol

## Abstract

**Introduction:**

Migraine is a common clinical disorder, ranks as the second most disabling disease worldwide, and often manifests with unilateral onset. Contralateral acupuncture (CAT), as a classical acupuncture method, has been proven to be effective in the treatment of migraine without aura (MWoA). However, its neural mechanisms have not been investigated using multimodal magnetic resonance imaging (MRI).

**Methods and analysis:**

In this multimodal neuroimaging randomized trial, a total of 96 female MWoA participants and 30 female healthy controls (HCs) will be recruited. The 96 female MWoA participants will be randomized into three groups: Group A (CAT group), Group B [ipsilateral acupuncture (IAT) group], and Group C (sham CAT group) in a 1:1:1 allocation ratio. Each group will receive 30 min of treatment every other day, three times a week, for 8 weeks, followed by an 8-week follow-up period. The primary outcome is the intensity of the migraine attack. Data will be collected at baseline (week 0), at the end of the 8-week treatment period (weeks 1–8), and during the 8-week follow-up (weeks 9–16). Adverse events will be recorded. Multimodal MRI scans will be conducted at baseline and after 8-week treatment.

**Discussion:**

This study hypothesized that CAT may treat MWoA by restoring pathological alterations in brain neural activity, particularly by restoring cross-integrated functional connectivity with periaqueductal gray (PAG) as the core pathological brain region. The findings will provide scientific evidence for CAT in the treatment of MWoA.

**Ethics and dissemination:**

The Medical Ethics Committee of the Second Affiliated Hospital of Yunnan University of Chinese Medicine has given study approval (approval no. 2022-006). This trial has been registered with the Chinese Clinical Trials Registry (registration no. ChiCTR2300069456). Peer-reviewed papers will be used to publicize the trial’s findings.

**Clinical trial registration:**

https://clinicaltrials.gov/, identifier ChiCTR2300069456.

## Introduction

Migraine is a common clinical neuro-vascular disorder that can affect individuals of any age. It is primarily characterized by recurrent episodes of severe throbbing headaches, often accompanied the symptoms such as nausea, vomiting, photophobia, and phonophobia (Bes et al., 2018). Migraine without aura (MWoA) is the most prevalent type, accounting for approximately 80% of cases (Bes et al., 2018). Studies (May and Schulte, 2016; GBD 2016 Headache Collaborators, 2018) have reported a global migraine prevalence of about 14.4%, with an estimated 3% of episodic migraines progressing to chronic migraines each year. Migraine has been recognized by the World Health Organization as one of the major neurological disabling diseases that imposes a substantial social burden (GBD 2016 Disease and Injury Incidence and Prevalence Collaborators, 2017; Burch et al., 2019; Peters, 2019). It ranks second only to stroke in terms of disability-adjusted life years lost (GBD 2015 Disease and Injury Incidence and Prevalence Collaborators, 2016).

The analgesic effect of acupuncture, the most widely used complementary alternative medicine method for MWoA, has gained international recognition. MWoA is now included in the recommended spectrum of diseases treated with acupuncture (Millstine et al., 2017). Numerous international clinical studies have demonstrated the effectiveness of acupuncture in preventing the recurrence of MWoA, reducing the number of days of migraine attacks (Zhao et al., 2017), alleviating the severity of pain attacks (Li et al., 2009), and improving patients’ quality of life (Yang et al., 2011). Furthermore, comprehensive guidelines for acupuncture-based migraine treatment have been developed in China (Jiao et al., 2016), and the clinical efficacy of acupuncture for prophylactic treatment of MWoA has been confirmed with minimal side effects (Linde et al., 2016; Zhao et al., 2017).

With the widespread application of magnetic resonance imaging (MRI) in migraine research and acupuncture’s mechanism, multiple studies (Russo et al., 2012; Tessitore et al., 2013; Li K. et al., 2015) have shown that migraine attacks are associated with abnormalities in the resting-state brain’s functional network connectivity. These abnormalities in brain function in migraine patients result from the cumulative effects of repeated pain episodes over an extended period of time (Goadsby et al., 2017). Consequently, investigating the pathogenesis of migraine and the mechanisms of acupuncture intervention at the MRI level has become a current research focus. Several ongoing studies aim to elucidate the functional brain mechanisms of acupuncture for migraine (Cheng et al., 2022; Hong et al., 2022). Some studies have confirmed that specific brain regions affected by acupuncture for MWoA, when compared to sham acupuncture, include the precentral gyrus, the postcentral gyrus, the hippocampus, the posterior cingulate gyrus, and the prefrontal cortex (Li K. et al., 2015). In comparison to acupuncture at non-meridian and non-acupoints, elevated blood oxygen level-dependent (BOLD) signals were observed in the orbitofrontal cortex, bilateral medial ventral part of the head of the medulla oblongata, the trigeminal complex, bilateral anastomosing midbrain, and the precentral gyrus (Li et al., 2017). Conversely, decreases were observed in the precuneus, the middle frontal gyrus, the posterior cingulate cortex, and the postcentral gyrus (Zhao et al., 2014; Qin et al., 2019). Regulation of brain functional activity in the posterior central gyrus may be one of the mechanisms of acupuncture in the treatment of MWoA (Frot et al., 2013).

Contralateral acupuncture (CAT), a classical acupuncture therapy, is widely used in the treatment of unilateral pain disorders and has demonstrated superior clinical efficacy (Whan et al., 2012; Gao et al., 2013). Migraine, characterized by predominantly one-sided pain, is a well-suited candidate for CAT. Our previous studies also support that CAT is effective in both acute and chronic migraine (Li et al., 2021, 2022, 2023; Huang et al., 2023). However, research on the brain function mechanism of acupuncture for migraine has mainly focused on unilateral or bilateral acupoints, with limited exploration of CAT. Studies on central brain mechanisms have indicated that needling acupoints on the left and right sides can yield different responses in terms of central function. For instance, needling the Yanglingquan acupoint on the left and right sides in healthy subjects resulted in significant differences in the regulation of various brain regions (Yeo et al., 2016). When comparing CAT and IAT for frozen shoulder treatment, CAT at the “Tiaokou” acupoint led to more pronounced pain reduction and improved shoulder joint function, and functional brain imaging revealed significant differences in the degree centrality of various brain regions, such as the cerebellum, thalamus, and posterior central gyrus (Yan et al., 2020), as well as differences in regional homogeneity (Zhang et al., 2018). Therefore, it is essential to explore the central neurological mechanism of CAT in the context of migraine.

The periaqueductal gray (PAG), a central structure in pain processing, forms a ring-shaped region with midbrain parietal dense neuronal nuclei. As a central brain region for pain modulation, the PAG is a focal point in functional magnetic resonance studies of acupuncture analgesia (Kong et al., 2010; Solstrand Dahlberg et al., 2018). Recent studies (Ito et al., 2016; Chen et al., 2017; Yu et al., 2021) have revealed altered functional structures within the PAG in migraine patients, disrupting upstream and downstream pain regulatory systems. We propose that the central mechanism of CAT for migraine is closely related to the PAG’s role in information transmission. Given the effectiveness of CAT in MWoA treatment, we have chosen the PAG as a region of interest (ROI) to explore how CAT influences brain functional connectivity (FC) in MWoA patients.

Hence, we designed this multimodal brain imaging trial with two aims: (1) to re-evaluate the efficacy of CAT in the treatment of MWoA by comparing CAT with sham CAT and ipsilateral acupuncture (IAT); and (2) to investigate the neural mechanism of CAT in treatment of MWoA by examining differences in the modulatory effects of CAT on the migraine pathology network compared to sham CAT and IAT through the integration of multimodal MRI. The findings of the study may investigate that the analgesic message of CAT can be delivered through one or more analgesic pathways centered on the PAG, and could provide high-quality evidence for the functional brain mechanisms of CAT for MWoA, as well as help provide valuable scientific support for clinical decision-making in the choice of CAT or IAT in the clinical acupuncture treatment of MWoA.

## Intervention and assessment tools

The materials and equipment involved in this study include acupuncture needles that will be used during treatment and non-penetrating retractable blunt needles used in the control group, routinely sterilized 75% alcohol and cotton swabs, and the Park sham acupuncture device (PSD) for blinding. MRI data will be acquired using a resonance scanner with a 16-channel phased-array head coil (3.0T GE Discovery 750; Milwaukee, WI, USA). Specific operational use is described in the Interventions and MRI data sections in the Methods section below.

## Methods

### Study design

**FIGURE 1:**
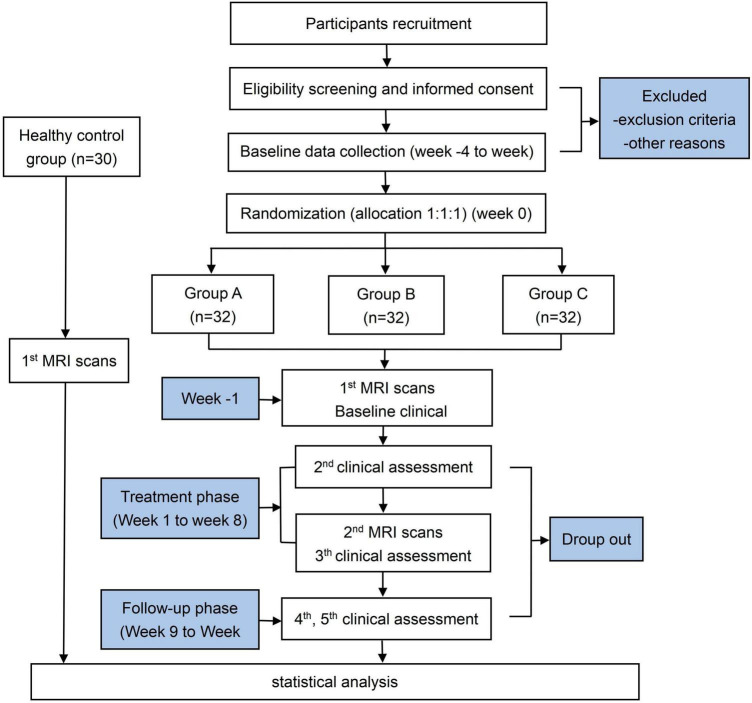
Flowchart of the research procedure.

This randomized clinical trial was designed to evaluate the treatment effect and neurological mechanisms of CAT on MWoA. A total of 96 female MWoA participants and 30 female healthy controls (HCs) will be enrolled. The study will last 20 weeks including a 4-week screening phase (week −4 to week 0), an 8-week treatment period (weeks 1–8), and an 8-week follow-up period (weeks 9–16). The enrollment schedules, interventions, and assessments are summarized in Table 1. During the screening phase, participants will be assessed headache diaries. Clinical outcomes will be evaluated after treatment and during the follow-up. Multimodal MRI scans will be performed at baseline and after treatment. A study flowchart is presented in Figure 1.

**TABLE 1:** Study schedule for data measurements.

	Study period					
	Enrolment	Allocation	Treatment phase		Follow-up	
Timepoint	−4 week	0 week	4 week	8 week	12 week	16 week
**Enrolment**						
Eligibility screen	×					
Informed consent	×					
Physical examination	×					
Randomization		×				
**Interventions**						
Group A (*n* = 32)			×	×	×	×
Group B (*n* = 32)			×	×	×	×
Group C (*n* = 32)			×	×	×	×
HCs group (*n* = 30)		×				
**Assessments**						
MRI parameters		×		×		
Headache diary	×	×	×	×	×	×
MSQ		×	×	×	×	×
MIDAS		×	×	×	×	×
HIT6		×	×	×	×	×
PSQI		×	×	×	×	×
SAS		×	×	×	×	×
SDS		×	×	×	×	×
Blinding/credibility test				×		
**Participants safety**						
AE			×	×	×	×
Laboratory test*		×		×		×

*The laboratory test includes complete blood count and differential count, absolute neutrophil count, aspartate aminotransferase, alanine aminotransferase, total bilirubin, blood urea nitrogen, creatinine, albumin, and coagulation test.

Group A, contralateral acupuncture (CAT) group; Group B, ipsilateral acupuncture (IAT) group; Group C, Sham CAT group; HCs, healthy controls; MRI, magnetic resonance imaging; VAS, visual analog scale; MSQ, Migraine-Specific Quality of Life Questionnaire; MIDAS, Migraine Disability Assessment; HIT6, Headache Impact Test-6; PSQI, Pittsburgh Sleep Quality Index; SAS, Self-Rating Anxiety Scale; SDS, Self-Rating Depression Scale; AE, adverse events.

This trial will be reported in accordance with the Standard Protocol Items: Recommendations for Intervention Trials (SPIRIT) guidelines (Chan et al., 2013; Supplementary File 1) and the Standards for Reporting Interventions in Clinical Trials of Acupuncture principles (STRICTA) (MacPherson et al., 2010). The protocol was approved by the Medical Ethics Committee of the Second Affiliated Hospital of Yunnan University of Chinese Medicine (2022-006) (Supplementary File 2) and is registered with the Chinese Clinical Trial Registry (registration no. ChiCTR2300069456).

### Recruitment and informed consent

All female MWoA participants and HCs will be recruited from the Second Affiliated Hospital of Yunnan University of Chinese Medicine and Kunming communities. Recruitment will take place through advertising from May 2023 to December 2024. Neurologists will strictly adhere to the inclusion and exclusion criteria to determine the eligibility of patients for this study. To facilitate recruitment, advertisements will be designed for both online platforms (e.g., WeChat public accounts and websites) and offline channels.

All participants will receive an explanation of the purpose and nature of this study protocol and they will be provided with comprehensive information about the study, including its procedures, potential benefits, and risks. Prior to enrollment, they will voluntarily sign an informed consent form (Supplementary File 3). All participants will have the option to withdraw from the study at any time without incurring penalties or losing benefits, and they are not required to provide a reason for their withdrawal. Any reasons for withdrawal will be documented.

### Participants

#### Inclusion criteria for patients with MWoA

(i) Meeting the diagnostic criteria for MWoA in the International Classification of Headache Disorders (ICHD-3) (Bes et al., 2018); (ii) right handed, female, high school or above education, aged 18–45 years old; (iii) in the past 3 months, the headache attack was ≥2 times per month, and the headache days were <15 days per month; (iv) moderate headache attacks (3 < visual analog scale (VAS) score < 7) (Lee et al., 2016) in the last 4 weeks (baseline period); (v) history of migraine for more than 1 year and no history of dysmenorrhea; (vi) no acupuncture or pharmacological treatment within 1 month; (vii) no metallic implants in the body and no MRI contraindications; and (viii) not participating in any other trials during the study period and signing a written informed consent form.

#### Exclusion criteria for patients with MWoA

(i) Bilateral or alternating unilateral migraine, or the presence of other chronic pain conditions (e.g., dysmenorrhoea, chronic low back pain, etc.); (ii) other types of headaches or headaches with an unknown diagnosis; (iii) presence of severe organ or physical illnesses, or severe mental health conditions such as anxiety, depression, insomnia, and other mental disorders; (iv) pregnancy, breastfeeding, or planning to give birth within the last year; (v) definite organic brain lesions or significant asymmetry between the right and left sides of the skull; (vi) contraindications to MRI (e.g., metallic foreign bodies, vascular stents, pacemakers, dentures, claustrophobia, etc.); (vii) current analgesic use or psychotropic drug dependence; (viii) populations with possible differences in brain structure and function, e.g., smoking or alcohol addictions; and (ix) participation in a similar study within the last 3 months.

#### Inclusion criteria for HCs

(i) Right handed, female, high school education or above, aged 18–45 years; (ii) no history of prolonged dysmenorrhoea, no neurological or psychiatric disorders, and no family history of a genetic predisposition; (iii) absence of physical abnormalities (e.g., cold, fever, etc.) during the trial; (iv) no medication or alcohol consumption at least 2 days prior to enrollment, and no use of stimulants for at least 1 month; and (v) willingness to participate and the ability to sign the informed consent form.

#### Exclusion criteria for HCs

Participants in the HCs group will exclude: (i) those with serious diseases of the heart, brain, liver, kidneys, and other vital organs; (ii) have a mental or physical disability; (iii) those with a suspected or actual history of alcohol or drug abuse; (iv) contraindications to MRI (e.g., metallic foreign bodies, vascular stents, pacemakers, dentures, claustrophobia, etc.); (v) pregnancy and breastfeeding; and (vi) populations with possible differences in brain structure and function, e.g., smoking or alcohol addictions.

### Randomization and blinding

All MWoA participants will be randomly assigned in a 1:1:1 ratio to one of three groups: Group A (CAT group), Group B (IAT group), and Group C (sham CAT group). Random numbers will be generated using a computer and sealed in opaque envelopes by an independent research assistant. After acceptance of the random allocation principle, participants will randomly select an opaque envelope to receive an allocation serial number, which will be recorded on a Case Report Form (CRF) by a dedicated research assistant. To maintain blinding, the researchers, assessors, and statistical analysts will be separated throughout the study. Due to the specific nature of the sham acupuncture operation, it will be difficult to blind the acupuncturists during the treatment. They will receive training from the investigator and will be instructed not to disclose the allocation of participants unless there are exceptional circumstances, such as severe infection or uncontrollable pain. During treatment, participants will be accommodated in separate rooms to minimize communication.

### Interventions

**FIGURE 2:**
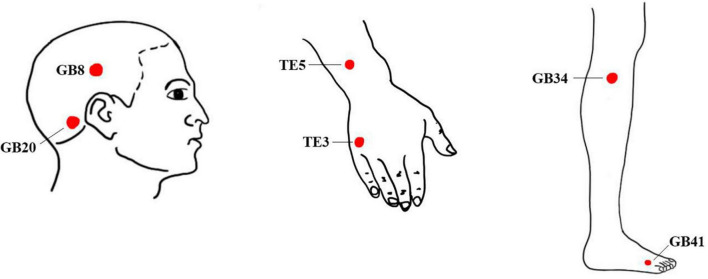
Location of acupoints.

Healthy controls will not undergo any treatment during the trial period, brain imaging data will be collected only once. Based on Traditional Chinese Medicine (TCM) theory, which suggests that most migraines are associated with temporal headaches, and following the findings from our previous studies on acupuncture for MWoA (Li et al., 2022, 2023), we have selected six frequently used acupoints along the Shaoyang meridian. These acupoints include Fengchi (GB20), Shuaigu (GB8), Waiguan (TE5), Zhongzhu (TE3), Yanglingquan (GB34), and Zulinqi (GB41). The positioning of these acupoints will be determined according to the standards set by the WHO Standard Acupuncture Point Locations in the Western Pacific Region (2008) (WHO Regional Office for the Western Pacific, 2008). Locations of the acupoints are presented in Table 2 and Figure 2. The interventions are compliant with the Consolidated Standards of Reporting Trials (Schulz et al., 2010) and the Standards for Reporting Interventions in Clinical Trials of Acupuncture (MacPherson et al., 2002). Patients will be instructed to assume a prone position with the head and face down, thus obscuring their vision during the acupuncture procedure. Then, the acupuncturist will routinely disinfect the skin around the acupoints.

**TABLE 2:** Location of acupoints.

Acupoints	Location
GB20 (Fengchi)	In the anterior region of the neck, inferior to the occipital bone, in the depression between the origins of sternocleidomastoid and the trapezius muscles.
GB8 (Shuaigu)	On the head, directly superior to the auricular apex, 1.5 B-cun superior to the temporal hairline.
TE5 (Waiguan)	On the posterior aspect of the forearm, midpoint of the interosseous space between the radius and the ulna, 2 B-cun proximal to the dorsal wrist crease.
TE5 (Waiguan)	On the posterior aspect of the forearm, midpoint of the interosseous space between the radius and the ulna, 2 B-cun proximal to the dorsal wrist crease.
GB34 (Yanglingquan)	On the fibular aspect of the leg, in the depression anterior and distal to the head of the fibula.
GB41 (Zulinqi)	On the dorsum of the foot, distal to the junction of the bases of the fourth and fifth metatarsal bones, in the depression lateral to the fifth extensor digitorum longus tendon.

### CAT group (Group A)

**FIGURE 3:**
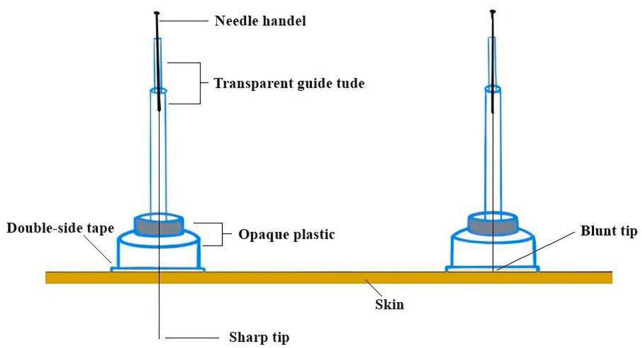
Park sham acupuncture device.

Patients in this group will receive acupuncture treatment at the acupoints on the contralateral side of the headache. The acupuncture [0.30 × (25 mm, 40 mm) Hua Tuo, Suzhou, China] will be used in this group. The PSD (see Figure 3; Li et al., 2022) will be fixed to the acupoints and the direction of needling is oblique downwards at GB 20, the level at GB 8, and straight at the rest of the acupoints. Due to the presence of hair on the head, the PSD may not adhere easily, so the needle will be secured in place with a hair patch. After needling the acupoints, the needle will be twisted 90–180° and lifted and inserted by 3–5 mm to induce a *deqi* sensation. The needles will be retained for 30 min, with needle manipulations performed every 15 min during this period.

### IAT group (Group B)

Patients in this group will receive acupuncture treatment at acupoints on the same side as their headache, following the same procedures as Group A.

### Sham CAT group (Group C)

This group will receive treatment with non-penetrating retractable blunt needles [0.30 × (25 mm, 40 mm) Hua Tuo, Suzhou, China]. During the procedure, the blunt needle will retract the hollow shank when it touches the skin, without piercing the skin (see Figure 3). However, patients will experience the sensation of being pricked by the needle. The blunt needle will also be secured with a hair patch at the head acupoints (Li et al., 2022). The needles will be retained for 30 min without any further manipulations.

All patients will receive 3 treatments per week, for a total of 24 treatments in the study. The operation will be performed by two acupuncturists with at least 3 years of clinical experience. Follow-up visits will be conducted every 4 weeks throughout the study, up to week 16.

### Urgent treatment with medication

Throughout the study, for the emergency treatment of acute migraine attacks in migraine patients, we will give the recommended NSAIDs for acute migraine attacks: ibuprofen (200–400 mg/times, not more than 800 mg/day) or diclofenac (50–100 mg/times, not more than 150 mg/day) according to the Chinese Migraine Diagnosis and Treatment Guidelines (2022 edition) (NBoCMD Association and HaSDSCotCRH Association, 2022), the types of drugs and dosage will be based on the patient’s needs and the doctor’s recommendation. However, they will not be allowed to use any prophylactic medications. Details such as the medication name, dosage, administration time, and the time taken for pain relief should be documented for any medication used.

### Sample size calculation

Regarding the sample size calculation for the modulation of brain functional networks by acupuncture, there is no established method. However, in line with the requirements of brain functional network analysis techniques (Bossier et al., 2020), a sample size of 30 cases per group should provide relatively stable reproducibility. We plan to include 30 cases in each treatment group, and with an estimated loss rate of 8%, we will aim for 32 cases per group, resulting in a total of 96 cases. The HCs group will consist of 30 cases, as they are undergoing examinations only once and are generally not participants in loss.

### Clinical outcome measures

#### Primary outcome

The primary outcome is the primary outcome is the intensity of the migraine attack, which will be assessed using a VAS score of 0–10. A headache diary form is designed to record details of each migraine attack, including its cause, duration, intensity, presence of aura, accompanying symptoms, frequency, and whether the migraine attack interfered with daily life and work, or emergency medication taken for unbearable pain. The headache diary form will be completed manually by the patient at the end of each migraine attack and will be monitored regularly by a dedicated researcher, who will collect and evaluate the data every 4 weeks (Supplementary File 4).

#### Secondary outcomes

Secondary outcomes include migraine attack frequency per month (−4 to 16 weeks), migraine attack days per month (−4 to 16 weeks), migraine attack intensity VAS score (8–16 weeks), duration of migraine (−4 to 16 weeks), emergency medication intake dose (−4 to 16 weeks), Migraine-Specific Quality of Life Questionnaire (MSQ) (−4 to 16 weeks), Migraine Disability Assessment Score (MIDAS) (−4 to 16 weeks), Headache Impact Test-6 (HIT6) (−4 to 16 weeks), Pittsburgh Sleep Quality Index (PSQI) (−4 to 16 weeks), Self-Rating Anxiety Scale (SAS) (−4 to 16 weeks), and Self-Rating Depression Scale (SDS), which are closely related to migraine. Blinding/reliability assessments will be conducted at the end of treatment (week 8) (Supplementary File 5), and treatment adherence evaluation will be assessed at the end of the follow-up period (week 16).

### MRI data

Magnetic resonance imaging data will be acquired within 1 week before the treatment and within 1 week after 8 weeks of treatment. The MRI will be acquired to avoid the patient’s migraine attacks, and all migraine patients should be without migraine (intermittent) for at least 72 h prior to the MRI scan before the scan is performed. Otherwise, the scan will be rescheduled. A Magnetic Resonance Scanner (3.0T GE Discovery 750; Milwaukee, WI, USA) with a 16-channel phased array head coil will be used, along with a restraining foam pad to minimize head motion and diminish scanner noise. The scans will be conducted at the Department of Radiology, the First Affiliated Hospital of Kunming Medical. Three-dimensional T1-weighted structural images will be obtained with the following scanning parameters: repetition time (TR) = 8.2 ms; echo time (TE) = 3.18 ms; field of view (FOV) = 256 mm × 256 mm; matrix = 512 × 512; in-plane resolution = 0.5 mm × 0.5 mm; slice thickness = 1 mm; 196 sagittal slices; flip angle = 9°. Resting-state functional MRI (rsfMRI) data will be collected within 7 min using the following parameters: TR = 2 s; TE = 30 ms; FOV = 240 mm × 240 mm; matrix = 64 × 64; in-plane resolution = 3.75 mm × 3.75 mm; slice thickness = 3.5 mm; 45 axial slices; flip angle = 90°, and 210 volumes will be acquired for each participant. Additional MRI data acquisition and preprocessing methods and procedures are described in Supplementary File 6.

### Seed-based FC analysis

The seed-based FC analysis will include all MWoA and HC participants and who completed the entire trial. PAG plays a pivotal role in the context of acupuncture for migraine treatment, so the PAG was chosen as the ROI for FC analysis. The location of the PAG seed will be selected based on previous studies (MNI coordinates: 0, −32, −12; size: 6 mm radius sphere) (Kucyi et al., 2013; Coulombe et al., 2017). The time course of each voxel in PAG will be extracted and averaged. Pearson correlation coefficients will be calculated between the average time series of PAG and each voxel throughout the whole brain. The obtained correlation coefficients will undergo Fisher-Z-transformed to increase normality. A clusterwise family-wise error rate (FWE) correction will also be considered.

### Voxel-base morphometry analysis

The gray matter volume (GMV) will be calculated using voxel-base morphometry (VBM) analysis in the FMRIB software library (FSL) (Jenkinson et al., 2012) version 5.0.9.^1^ First, all T1-weighted data will undergo brain extraction using the Brain Extraction Tool (BET) (Smith, 2002) and will be visually checked by an experienced neurologist to remove any leftover non-brain tissue. Second, using the FMRIB’s Automated Segmentation Tool (FAST) (Smith, 2002), the obtained brain-extracted data will be segmented into gray matter (GM), white matter (WM), and cerebrospinal fluid (CSF). Third, the resulting GM images were non-linearly registered to the GM ICBM-152 template using the non-linear registration tool FNIRT, and then averaged and flipped along the x-axis to obtain a first-pass, study-specific GM template. Next, the native segmented GM images will be nonlinearly re-registered to the above GM template and averaged to generate the final symmetric, study-specific GM template. Furthermore, the registered GM images will undergo modulation using the Jacobian of the warp field to produce maps of GMV. Finally, the obtained GMV images will be smoothed with a Gaussian kernel of 3 mm (a full-width half-maximum of ∼7 mm).

### Clinical data analysis

Statistical analysis will be performed by independent statisticians who will have no knowledge of group allocation or the intervention process. Clinical outcomes will be analyzed using SPSS 28.0 statistical software (SPSS Inc., Chicago, IL, USA). All statistical analyses will adhere to the intention-to-treat principles. The missing data of participants who drop out will be handled using multiple imputation by chained equations (Azur et al., 2011), which can impute mixtures of continuous, binary, unordered categorical, and ordered categorical data. In addition, a per-protocol population analysis will be performed on participants who have completed at least 80 percent of their treatment protocol after randomization. Qualitative data will be presented as percentages [*n* (%)] and compared using the Chi-square (χ^2^) test. Quantitative data will be reported as means ± SDs and IQRs. Shapiro–Wilk tests will be performed to assess the normality of the data. Comparisons among the three groups will be analyzed using one-way analysis of variance (ANOVA), and non-parametric tests will be employed when the distribution is not normal. Outcomes will be analyzed using a one-way repeated measures ANOVA to evaluate the impact of time, group, and time × group interactions on migraine attacks. Comparisons of categorical data among groups will be examined using χ^2^ tests or Mann–Whitney U tests. All hypothesis tests will be two-tailed analyses, with statistical significance determined by a *p*-value < 0.05.

### Neuroimaging data analysis

The structural MRI data will be analyzed using the VBM toolbox within SPM12. The steps will include interlaminar time difference correction, spatial difference correction, head motion correction, normalization, and Gaussian smoothing (Yao et al., 2021). During head motion correction, images of participants with translations of less than 1 mm and rotational movements of less than 2° will be involved in the subsequent analysis. The MRI data will be analyzed using PANDA, GRETNA and BrainNet Viewer toolboxes and SurfStat. In VBM analysis, generalized linear regression model analysis will be used and between-group VBM comparisons will be made using corrected subject migraine attack intensity, migraine attack frequency, etc. (control covariates: age, PSQI, SAS, SDS, etc.) and total intracranial volume by two-sample *t*-test. A significant difference between groups will be indicated when *p* < 0.05 (FWE corrected). Only collections with more than 20 voxels will be considered as statistically significant brain regions. First, the FC of PAG in the baseline session will be compared between the migraine patients and normal participants using two sample *t*-test with FWE correction. Second, two-factor three-level mixed design ANOVA (Time: baseline and after treatment; Treatment: CAT, IAT, and sham CAT) will be conducted on migraine patients to explore the effect of treatment schedule on the FC of PAG after FWE correction. *Post hoc* analysis will be implemented using paired *t*-test to investigate the alteration of FC between before and after treatment for each treatment schedule. Finally, Pearson correlation analysis will be performed between the change in VAS score and the changes of FC in significant regions for three treatment schedules, respectively. The statistical analysis for the VBM will follow a similar procedure to that for the FC of PAG.

### Safety assessments

During the baseline period, all participants will undergo routine blood, urine, and stool tests, as well as electrocardiograms. Adverse events (AEs) encompass those related to acupuncture treatment, such as bleeding, hematoma, pain, and syncope, as well as symptoms potentially resulting from drug use, including headache, nausea, and vomiting. These incidents will be meticulously documented on the CRFs when they occur. In cases of serious adverse events, such as death, life-threatening disability, or hospitalization, the study leader will promptly report these occurrences to both the site and the hospital’s ethics committee within 24 h and ensure their accurate inclusion in the CRF. All AEs will be diligently followed up until they are appropriately addressed. Decisions regarding the potential termination of the trial will be made by the research ethics committee if deemed necessary.

### Data management and quality control

To ensure the study’s smooth progress, the research leader will provide training to all staff, covering the project’s operational aspects, observation indicators, and participant criteria. MRI data collection will follow specific procedures. The research leader will oversee the study to maintain data accuracy. CRFs and paper data will be securely stored, and important documents will be retained for 5 years post-publication. Protocol deviations will be reported to the Ethics Committee, which can decide on changes or trial termination.

### Anticipated results

**FIGURE 4:**
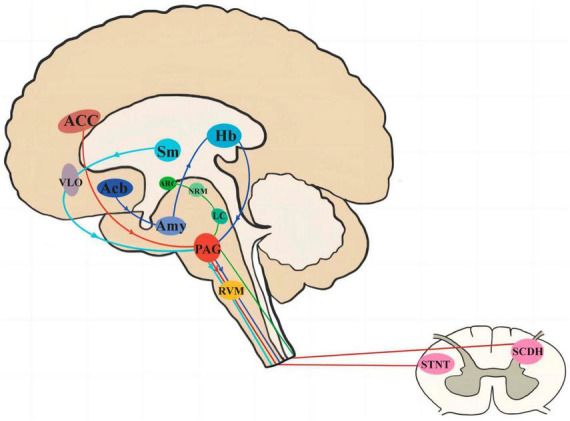
The pathway of PAG involved in pain signal transduction. PAG, periaqueductal gray; ACC, anterior cingulate cortex; RVM, rostral ventral medulla; SCDH, spinal cord dorsal horn; STNT, spinal tract nucleus of the trigeminal nerve; Acb, nucleus accumben; Amy, amygdala; Hb, habenular nucleus; ARC, arcuate nucleus; NRM, nucleus raphe medius; LC, locus coeruleus; Sm, thalamic nucleus submedius; VLO, ventrolateral orbital cortex.

Periaqueductal gray plays a bi-directional role in both upward and downward projections, and is involved in the regulation of several analgesic pathways, including four main ones (see Figure 4): (1) The brainstem downward inhibitory system, i.e., the anterior cingulate cortex (ACC)-PAG-rostral ventral medulla (RVM)-spinal cord dorsal horn (SCDH)/spinal tract nucleus of the trigeminal nerve (STNT) pathway (Heinricher et al., 2009; Kong et al., 2010).(2) Midbrain limbic analgesic circuit, i.e., nucleus accumben (Acb)-amygdala (Amy)-habenular nucleus (Hb)-PAG-RVM (Napadow et al., 2007; Fang et al., 2009); (3) arcuate nucleus (ARC)-nucleus raphe medius (NRM)-locus coeruleus (LC) – PAG – SCDH pathway (Longchuan et al., 1988); and (4) Thalamic nucleus submedius (Sm)-Ventrolateral orbital cortex (VLO)-PAG-spinal cord loop (Jingshi and Bin, 2002). The results of this study will elucidate the central mechanism of contralateral acupuncture for migraine, whereby analgesic messages from CAT may be delivered simultaneously through one or more analgesic pathways of PAG transmission.

## Discussion

In recent years, MRI techniques have played a crucial role in advancing our understanding of the mechanisms underlying migraine. Studies have shown that MWoA patients exhibit notable pathological changes in specific brain regions and alterations in functional network connections, particularly involving the brainstem, brain bridge, and thalamus (Wei et al., 2019; Qin et al., 2020), and there are differences in functional brain activities during the ictal and interictal phases in the brainstem, brain bridge, thalamus, insula, cerebellum, and cingulate gyrus (Maleki et al., 2021). The severity of migraine symptoms has been found to be positively correlated with the strength of connections in the hypothalamus and inversely correlated with connections in the prefrontal cortex (Coppola et al., 2020). These structural and functional irregularities within pathological brain regions and networks associated with migraine are linked to overall dysregulation in the integration and processing of pain components during migraine attacks (David et al., 2020).

Periaqueductal gray plays a pivotal role in the context of acupuncture for migraine treatment. PAG releases various substances involved in pain regulation. Acupuncture modulates PAG-related pathways, such as PAG-RVM (Ma et al., 2021). Also, percutaneous auricular vagus nerve stimulation at 1 and 20 Hz affects PAG’s functional connectivity differently (Cao et al., 2021). A previous study by our team (Li et al., 2016) also found that migraine patients had reduced PAG connectivity with the anterior cingulate cortex/medial prefrontal cortex (rACC/mPFC) associated with increased pain. After acupuncture treatment targeting Shaoyang and Yangming meridians, PAG connectivity with rACC decreased, but connectivity between PAG, rACC, and the ventral striatum (VST) increased, correlating with pain relief. Therefore, using PAG as an ROI seed for CAT’s effect on brain connectivity in MWoA patients can shed light on CAT’s central mechanisms in treating MWoA.

In this trial, according to previous clinical experience (Li et al., 2021, 2023), we selected a fixed acupuncture prescription consisting of GB20, GB8, TE5, TE3, GB34, and GB41. Furthermore, previous research revealed gender differences in brain functional network abnormalities among migraine patients (Liu et al., 2011, 2012; Maleki et al., 2012) with females showing higher abnormalities. Female migraineurs exhibit greater brain activation responses in certain brain regions (e.g., amygdala, basal ganglia, and posterior cingulate cortex), which are involved in the processing of the emotional aspects of pain, when compared to males who suffer from migraines (Maleki et al., 2012). Therefore, selecting only female migraine patients will help to eliminate sex-induced differences in brain function. The IAT group was established to validate the comparative efficacy of acupuncture treatment based on acupoint selection on the side of the headache versus the contralateral side of the headache.

Although some studies suggested that CAT treatment is more effective than IAT, the quality of these studies is generally low (Hong et al., 2009; Li Y. et al., 2015). Further research is needed to confirm the superiority of CAT. Additionally, to minimize potential confounding factors in functional brain imaging analysis, we included the sham CAT group. Migraine sufferers often experience comorbid psychiatric disorders, particularly anxiety and depression (Antonaci et al., 2011; Minen et al., 2016), which can influence migraine’s prevalence, treatment outcomes, and prognosis (Louter et al., 2015). Some previous studies on central mechanisms of migraine have also included insomnia, anxiety, and depression as secondary outcomes for evaluation (Tu et al., 2019; Xu et al., 2023). In our study, we used these scales as secondary measures to account for anxiety and depression, ensuring these factors do not confound our results.

This study has several strengths. First, this study utilizes the ancient acupuncture method CAT to explore its neural mechanisms in treating MWoA through multimodal MRI. The results may provide further evidence for the selection of acupuncture on the contralateral or ipsilateral side for treating MWoA, for the promotion of more acupuncture therapies for migraine, and the development of the analgesic mechanism of acupuncture. Second, the study compares the efficacy of CAT and IAT, providing insights into the differences between right and left acupoint acupuncture and the brain’s cross-regulatory mechanisms. Third, to explore the brain effect mechanism of acupuncture, the study concentrates on unilateral onset headache cases, excluding the influence of multiple meridian identification and acupoint selection. Fourth, to ensure reliable results, a sham acupuncture group is included, participant groups are kept separate, and outcome assessors and statisticians remain blind to group assignments, minimizing potential bias.

This study has several limitations worth mentioning. First, due to the specificity of the acupuncture manipulation, the sham CAT group may cause a wide range of peripheral, segmental, and central physiological responses of unpredictable magnitude even without inserting blunt needles subcutaneously. Second, achieving blinding in the CAT and IAT groups is challenging due to the differences in acupoints. Furthermore, acupuncturists are aware of the subgroups, potentially transferring their attitudes in favor of or against the intervention to the participants. The lack of blinding of the acupuncturists, the use of self-reported outcomes, and the possible placebo effects of acupuncture may potentially bias study results. Third, the study will include only one center, but it is replicable and the results of its trials can be evaluated later through multiple centers.

## Conclusion

In conclusion, our trial aimed to demonstrate the central cross-integration mechanism of the classical acupuncture method CAT for MWoA. By further verifying whether the efficacy of CAT for MWoA is superior to that of IAT or sham CAT, as well as the respective differences in FC information through regulating the PAG’s own subgroup network and with external analgesic networks, the findings will provide high-quality evidence for the elaboration of the functional brain mechanisms of CAT in the treatment of MWoA. This research can contribute to the advancement of acupuncture analgesia mechanisms and provide valuable scientific support for clinical decision-making between CAT and IAT for MWoA treatment.

## Data availability statement

The original contributions presented in this study are included in the article/Supplementary material, further inquiries can be directed to the corresponding authors.

## Ethics statement

The studies involving humans were approved by the Medical Ethics Committee of the Second Affiliated Hospital of Yunnan University of Chinese Medicine (2022-006). The studies were conducted in accordance with the local legislation and institutional requirements. The participants provided their written informed consent to participate in this study.

## Author contributions

ZC: Conceptualization, Writing – original draft. QL: Conceptualization, Writing – review & editing. YL: Data curation, Formal analysis, Writing – review & editing. GH: Conceptualization, Methodology, Writing – review & editing. YH: Methodology, Writing – review & editing. XP: Investigation, Resources, Writing – review & editing. YG: Investigation, Resources, Writing – review & editing. BZ: Supervision, Writing – review & editing. XT: Investigation, Resources, Writing – review & editing. ZL: Funding acquisition, Investigation, Resources, Writing – review & editing. TG: Conceptualization, Project administration, Writing – review & editing. FL: Conceptualization, Writing – review & editing.

## Footnotes

1
http://www.fmrib.ox.ac.uk/fsl/

## Supplementary material

Supplementary File 1 SPIRIT_Fillable-checklist-15-Aug-2013.

Supplementary File 2 Ethical approval documentation.

Supplementary File 3 Informed consent of participants.

Supplementary File 4 Headache diary.

Supplementary File 5 Blinding questionnaire.

Supplementary File 6 Magnetic resonance imaging data acquisition and preprocessing.
