# Supplementary material for: Contralateral acupuncture for migraine without aura: a randomized trial protocol with multimodal MRI
Source: Front Neurosci. 2024 Mar 15;18:1344235. doi: 10.3389/fnins.2024.1344235 (PMC10979701; doi:10.3389/fnins.2024.1344235)
Supplement: Supplementary File 1 — SPIRIT_Fillable-checklist-15-Aug-2013. [file Data_Sheet_1.zip › Data_Sheet_1/Data Sheet 1.pdf]

Headache Diary

Name: _______________________

Address: _____________________

Tel No: ______________________

Book No.______________________

[Instructions for filling in]

Your headache diary is used to record all symptoms related to your headache. It does not take much of your time to keep a headache diary, you answer these questions after each headache attack and you need to record all the symptoms you experience for 4 weeks, please try to be as accurate and complete as possible. Your headache diary will help your doctor to decide on the correct treatment strategy. Your symptoms may not be obvious during this time, but as you record each symptom, the pattern of your symptoms and the progression of your condition over time will give a strong indication of your condition. If you have any questions, please call:___________or your doctor, or you can e-mail:____________.

Doctor in charge:______________.

Doctor in charge Tel;_____________.

Note 1:

Level of severity: 0 = no pain; 1 (mild pain) = no effect on daily activities; 2 (moderate pain) = effect on daily activities; 3 (severe pain) = unable to perform daily activities

Note 2:

VAS reading: Please read out the number of your headache in terms of severity.

No pain |—|—|—|—|—|—|—|—|—|—| Maximum pain

0 1 2 3 4 5 6 7 8 9 10

0: no pain;

1-3: mild pain, not affecting work or life;

4-6: moderate pain, affecting work and life;

7-10: severe pain, severe pain, affecting work and life.

Note 3:

If the headache is still on when you go to sleep the day before and disappears when you wake up the next morning, the end time of the headache is recorded as the waking time when you wake up the next day; if the headache is longer than 0.5 hours per day, it is calculated as 1 day.

Note 4:

The time should be recorded in a 24-hour format:_____hours_____ minutes.

Note 5:

The name of the medication to be recorded, ____tablets/time,____ times/day,____days

Note 6:

Headache causes (including emotions or stress, sleep, environment, food and drink, medication,

menstruation, lifestyle, other)

Note 7:

Please fill in your menstrual period start and end dates: _____to ______

Date / Items					
The lasting time of headache (start time-end time)					
Aura (1. none; 2. visual aura; 3. other aura)					
Headache Causes					
Headache location (left side, right side)					
Level of headache severity (0-3) (0 none; 1 mild; 2 moderate; 3 severe)					
Headache severity VAS score (0-10)					
Associated symptoms and severity	Nausea (0 none; 1 mild; 2 moderate; 3 severe)				
	Vomiting (0 none; 1 mild; 2 moderate; 3 severe)				
	Photophobia (0 none; 1 mild; 2 moderate; 3 severe)				
	Photophobia (0 none; 1 mild; 2 moderate; 3 severe)				
	Photophobia (0 none; 1 mild; 2 moderate; 3 severe)				
Whether daily work is affected by headache (No□; Yes□). If yes, please write the time affected (hours, h)		No□；Yes□ （ ）h	No□；Yes□ （ ）h	No□；Yes□ （ ）h	No□；Yes□ （ ）h
During acute attacks: taking pain medication/ acupuncture	Name of medication used for treatment				
	Dose, number of times				
	Headache half an hour after treatment	Disappear□ Relieved□ No change□	Disappear□ Relieved□ No change□	Disappear□ Relieved□ No change□	Disappear□ Relieved□ No change□
	Headache 2 hours after treatment	Disappear□ Relieved□ No change□	Disappear□ Relieved□ No change□	Disappear□ Relieved□ No change□	Disappear□ Relieved□ No change□
Adverse effects during treatment and ways to relieve them					

头 痛 日 记

姓名：____________________

住址：____________________

电话：____________________

第____册

[填写说明]

您的头痛日记用于记录与头痛有关的所有症状。记录头痛日记不需要占用您太多时间，您在每次头痛发作后回答这些问题，需要记录4周您观察到的所有症状，请尽量保证准确性、完整性。您的头痛日记有助于您的医生制定出正确的治疗策略。在这段时间内您的症状可能不明显，但是您将每一种症状都记录下来后，您的症状模式和病情随时间变化发展的状况都将有力地说明您的病情。如有任何问题，请致电：____________或者您的主管医生，也可以发送E－mail至：_____________。

主管医生：_________________

主管医生电话：________________

说明1：

程度分级：0=无痛；1（轻度疼痛）＝不影响日常活动；2（中度疼痛）＝影响日常活动；3（重度疼痛）＝不能进行日常活动

说明2：

VAS读数 ：请读出您头痛轻重数字

不痛 |—|—|—|—|—|—|—|—|—|—| 最痛

0 1 2 3 4 5 6 7 8 9 10

0：无痛；

1-3：轻度疼痛，不影响工作和生活；

4-6：中度疼痛，影响工作，不影响生活；

7-10：重度疼痛，疼痛剧烈，影响工作和生活。

说明3：如果是前一天入睡时仍觉头痛，第二天晨醒时头痛消失，则头痛结束时间计为第二天睡醒起床时间；如果每天头痛的时间大于0.5小时，计算为1天

说明4：时间填写格式按24小时制：　　时　　分

说明5：药物服用情况记录需填写药物名称，？片/次， ？次/天，共服用 ？天

说明6：头痛诱因（包括：情绪或压力，睡眠，环境，食品饮料，药物，月经来潮，生活方式，其他）

说明7：请填写您的月经起止时间：____________至____________

项目 \ 日期					
头痛持续时间（开始时间～结束时间）					
先兆（1.无；2.视觉先兆；3.其他先兆）					
头痛诱因					
头痛部位（左侧，右侧）					
头痛程度分级（0-3）<br>（0无；1轻度；2中度；3重度）					
头痛程度VAS读数（0-10）					
伴随症状及程度	恶心（0无；1轻度；2中度；3重度）				
	呕吐（0无；1轻度；2中度；3重度）				
	畏光（0无；1轻度；2中度；3重度）				
	怕吵（0无；1轻度；2中度；3重度）				
	其他（0无；1轻度；2中度；3重度）				
头痛时日常生活工作是否受影响（否☐；是☐）<br>如为是，请填写受影响的时间（小时,h）		否☐；是☐<br>（　　）h	否☐；是☐<br>（　　）h	否☐；是☐<br>（　　）h	否☐；是☐<br>（　　）h
急性发作时：服用止痛药物/针刺	治疗药物名称				
	剂量、次数				
	治疗后半小时头痛情况	消失☐<br>缓解☐<br>无变化☐	消失☐<br>缓解☐<br>无变化☐	消失☐<br>缓解☐<br>无变化☐	消失☐<br>缓解☐<br>无变化☐
	治疗后2小时头痛情况	消失☐<br>缓解☐<br>无变化☐	消失☐<br>缓解☐<br>无变化☐	消失☐<br>缓解☐<br>无变化☐	消失☐<br>缓解☐<br>无变化☐
治疗期间的不良反应及缓解方式					
