# Supplementary material for: Contralateral acupuncture for migraine without aura: a randomized trial protocol with multimodal MRI
Source: Front Neurosci. 2024 Mar 15;18:1344235. doi: 10.3389/fnins.2024.1344235 (PMC10979701; doi:10.3389/fnins.2024.1344235)
Supplement: Supplementary File 1 — SPIRIT_Fillable-checklist-15-Aug-2013. [file Data_Sheet_1.zip › Data_Sheet_1/Data Sheet 2.pdf]

Blinding test

Do you think you were given acupuncture treatment or placebo acupuncture?	Yes ☐ No ☐
How sure are you on your answer on a scale of 0 to 10? (0 = very uncertain and 10 = completely certain)	________________

盲法测试

您认为您接受的针灸是真正的针灸吗？	是 ☐ 否 ☐
从 0-10，您对自己的答案有多确实？(0 =非常不确定，10 =完全确定)	________________
