# Supplementary material for: Contralateral acupuncture for migraine without aura: a randomized trial protocol with multimodal MRI
Source: Front Neurosci. 2024 Mar 15;18:1344235. doi: 10.3389/fnins.2024.1344235 (PMC10979701; doi:10.3389/fnins.2024.1344235)
Supplement: Supplementary File 1 — SPIRIT_Fillable-checklist-15-Aug-2013. [file Data_Sheet_1.zip › Data_Sheet_1/Data Sheet 3.pdf]

MRI data acquisition

MRI data will be acquired within 1 week before the treatment and within 1 week after 8 weeks of treatment. Magnetic We will use a Resonance Scanner (3.0T GE Discovery 750; Milwaukee, WI, USA) with a 16-channel phased array head coil, along with a restraining foam pad to minimize head motion and diminish scanner noise. The scans will be conducted at the Department of Radiology, the First Affiliated Hospital of Kunming Medical. 3D T1-weighted structural images will be obtained with the following scanning parameters: repetition time (TR) = 8.2 ms; echo time (TE) = 3.18 ms; field of view (FOV) = 256×256 mm2; matrix = 512×512; in-plane resolution = 0.5×0.5 mm2; slice thickness = 1 mm; 196 sagittal slices; flip angle = 9°. Resting-state functional MRI (rsfMRI) data will be collected within 7 minutes using the following parameters: TR = 2s; TE = 30 ms; FOV = 240×240 mm2; matrix = 64×64; in-plane resolution = 3.75×3.75 mm2; slice thickness = 3.5 mm; 45 axial slices; flip angle = 90°, and 210 volumes will be acquired for each participant.

To ensure participants remain awake during the rsfMRI scanning, they will be required to relax, stay still, keep their eyes open, and focus on a fixation cross. The fixation cross will be projected onto a screen positioned at the head of the magnet bore and viewed through a mirror attached to the head coil. After the rsfMRI scanning, participants will be asked if they were awake during the scanning, and all participants confirmed that they were awake. Considering the possibility of changes related to the menstrual cycle, participants will be obtained before or outside of their menstrual period. If any participant experiences discomfort, the scan will be terminated. A specialist staff member will perform a qualitative examination of the images to detect any brain lesions or structural abnormalities.

MRI data preprocessing

Preprocessing will be performed using the Statistical Parametric Mapping (SPM12, www.fil.ion.ucl.ac.uk/spm/software/spm12/) and the FC Toolbox (CONN version 17f,

https://www.nitrc.org/projects/conn). The first five volumes of the rsfMRI data for each session will be discarded to ensure signal stabilization and allow participants to acclimate to the scanning noise. Then, the remaining 205 volumes of the rsfMRI will be preprocessed with the CONN's default preprocessing pipeline which includes 8 steps. 1) Functional realignment and unwarp: The remaining rsfMRI data will be realigned to the middle volume of each session, correcting for head motion using the SPM12 realign & unwarp procedure (Andersson et al., 2001). To control the influence of the head motion on the results, participants with translations greater than 2 mm or rotations exceeding 2° during a session will be excluded. In total, six participants will be excluded due to excessive head motion. 2) Centering: The realigned functional data will be centered to (0,0,0) coordinates. 3) Slice-timing correction: To account for the sequential nature of the MRI acquisition protocol, the slice order of the rsfMRI will be temporally adjusted using the SPM12 slice-timing correction procedure (Henson et al., 1999). 4) Functional outlier detection: Functional scans will be screened for artifacts and motion outliers using the Artifact Detection Toolbox (ART; https://www.nitrc.org/projects/artifact_detect/) with intermediate settings (97th percentiles in normative sample). Timepoints will be flagged as outliers with global blood oxygenation level dependent (BOLD) signal changes exceeding 5 standard deviations and framewise displacement above 0.9 mm. 5) Functional direct segmentation and normalization: The slice-timing corrected rsfMRI data will be segmented into grey matter (GM), white matter (WM), and cerebrospinal fluid (CSF), and then normalized to the standard Montreal Neurological Institute (MNI) space using the default Tissue Probability Maps using SPM12 unified segmentation and normalization procedure (Ashburner and Friston, 2005), with a voxel size of $2 \times 2 \times 2$ mm^3. 6) Structural image centering: The 3D T1-weighted structural images will be centered to (0,0,0) coordinates. 7) Structural segmentation and normalization: The structural data will be segmented into GM, WM, and CSF, and then normalized into the standard MNI space with a voxel size of $2 \times 2 \times 2$ mm^3, following a similar procedure as

applied to the functional data. 8) Functional smoothing: To enhance the BOLD signal-to-noise ratio, the functional data will be spatially smoothed using a Gaussian kernel with 8 mm full width at half maximum.

Finally, the denoising pipeline will be applied to remove potential confounders from the BOLD signal using an anatomical component-based noise correction procedure (aCompCor) (Behzadi et al., 2007). The confounders consisted of 10 noise components from WM and CSF (five components each) with the principal component analysis, 12 motion-related parameters (3 translation and 3 rotation parameters plus their associated first-order derivatives), and scrubbing parameters obtained from the functional outlier detection. Then, temporal band-pass filtering will be implemented with a frequency window of 0.008–0.09 Hz to focus on slow-frequency fluctuations and minimize the influence of physiological, head-motion, and other noise sources. Additionally, the linear detrending will also be performed to remove linear trends within each functional session.

Andersson, J.L., Hutton, C., Ashburner, J., Turner, R..Friston, K. (2001). Modeling geometric deformations in EPI time series. *Neuroimage.* 13, 903-919. doi:10.1006/nimg.2001.0746

Ashburner, J..Friston, K.J. (2005). Unified segmentation. *Neuroimage.* 26, 839-851. doi:10.1016/j.neuroimage.2005.02.018

Behzadi, Y., Restom, K., Liau, J..Liu, T.T. (2007). A component based noise correction method (CompCor) for BOLD and perfusion based fMRI. *Neuroimage.* 37, 90-101. doi:10.1016/j.neuroimage.2007.04.042

Henson, R., Buechel, C., Josephs, O..Friston, K.J.N. (1999). The slice-timing problem in event-related fMRI. 9, 125-.
